# Electronic Word of Mouth on Twitter About Physical Activity in the United States: Exploratory Infodemiology Study

**DOI:** 10.2196/jmir.2870

**Published:** 2013-11-20

**Authors:** Ni Zhang, Shelly Campo, Kathleen F Janz, Petya Eckler, Jingzhen Yang, Linda G Snetselaar, Alessio Signorini

**Affiliations:** ^1^The University of Iowa alumnusIowa City, IAUnited States; ^2^The University of IowaDepartment of Community and Behavioral HealthIowa City, IAUnited States; ^3^The University of IowaDepartment of Health and Human PhysiologyIowa City, IAUnited States; ^4^University of StrathclydeSchool of HumanitiesGlasgowUnited Kingdom; ^5^Kent State UniversityDepartment of Social and Behavioral SciencesKent, OHUnited States; ^6^The University of IowaDepartment of EpidemiologyIowa City, IAUnited States; ^7^The University of IowaDepartment of Computer ScienceIowa City, IAUnited States

**Keywords:** Twitter messaging, social marketing, motor activity

## Abstract

**Background:**

Twitter is a widely used social medium. However, its application in promoting health behaviors is understudied.

**Objective:**

In order to provide insights into designing health marketing interventions to promote physical activity on Twitter, this exploratory infodemiology study applied both social cognitive theory and the path model of online word of mouth to examine the distribution of different electronic word of mouth (eWOM) characteristics among personal tweets about physical activity in the United States.

**Methods:**

This study used 113 keywords to retrieve 1 million public tweets about physical activity in the United States posted between January 1 and March 31, 2011. A total of 30,000 tweets were randomly selected and sorted based on numbers generated by a random number generator. Two coders scanned the first 16,100 tweets and yielded 4672 (29.02%) tweets that they both agreed to be about physical activity and were from personal accounts. Finally, 1500 tweets were randomly selected from the 4672 tweets (32.11%) for further coding. After intercoder reliability scores reached satisfactory levels in the pilot coding (100 tweets separate from the final 1500 tweets), 2 coders coded 750 tweets each. Descriptive analyses, Mann-Whitney *U* tests, and Fisher exact tests were performed.

**Results:**

Tweets about physical activity were dominated by neutral sentiments (1270/1500, 84.67%). Providing opinions or information regarding physical activity (1464/1500, 97.60%) and chatting about physical activity (1354/1500, 90.27%) were found to be popular on Twitter. Approximately 60% (905/1500, 60.33%) of the tweets demonstrated users’ past or current participation in physical activity or intentions to participate in physical activity. However, social support about physical activity was provided in less than 10% of the tweets (135/1500, 9.00%). Users with fewer people following their tweets (followers) (*P*=.02) and with fewer accounts that they followed (followings) (*P*=.04) were more likely to talk positively about physical activity on Twitter. People with more followers were more likely to post neutral tweets about physical activity (*P*=.04). People with more followings were more likely to forward tweets (*P*=.04). People with larger differences between number of followers and followings were more likely to mention companionship support for physical activity on Twitter (*P*=.04).

**Conclusions:**

Future health marketing interventions promoting physical activity should segment Twitter users based on their number of followers, followings, and gaps between the number of followers and followings. The innovative application of both marketing and public health theory to examine tweets about physical activity could be extended to other infodemiology or infoveillance studies on other health behaviors (eg, vaccinations).

##  Introduction

### Background

Twitter, a microblogging service and social networking site [1,2], provides a public platform to study the distribution and determinants of information with the ultimate aim to inform public health and public policy. This has been referred to as infodemiology or infoveillance with the primary aim of surveillance [3,4]. A few pioneering infoveillance scholars have successfully used Twitter to monitor people’s status updates to track illness over time, often referred to as syndromic surveillance [3-6], such as during the H1N1 outbreak [7,8]. Other infoveillance studies analyzed how people share health information on Twitter and have monitored their health-related behaviors [3,4], such as antibiotic use [9], drug abuse [10], dietary behavior [11], and smoking behavior [12]. However, to date little larger-scale research has addressed the distribution of information about health behaviors among personal Twitter users rather than organizational Twitter accounts in the United States.

The current exploratory study aims to fill this gap and to inform the development of future health marketing interventions aiming to promote physical activity in the United States on this far-reaching communication platform. The Pew Internet & American Life Project found that 13% of online adults [13] and 8% of teenagers (aged 12-17 years) use Twitter [14] in the United States. In 2009, 12% of the people who looked online for health information also used Twitter to share health updates about themselves or to see updates about others [15].

The current study focuses on the health behavior of physical activity, in part to address the epidemic of inactivity in the United States. According to the 2009 US Youth Risk Behavior Survey, only 18% of students in grades 9 to 12 participated in at least 60 minutes of physical activity per day, and only 33% attended physical education class daily [16]. An examination of physical activity prevalence in the United States, derived from the results of the 2008 National Health Interview Survey, found that fewer than half (43.5%) of adults were aerobically active, a little over one-fifth (21.9%) met the muscle-strengthening guideline, and only 18.2% met both the muscle-strengthening guideline and were aerobically active throughout the year [17].

The current study examines the dissemination and sharing of information about physical activity among personal Twitter users rather than organizational Twitter accounts, physical activity facility, or physical activity equipment company accounts. In marketing research, digital sharing among individuals is called electronic word of mouth (eWOM), which refers to the online information exchange between and among a large number of consumers about a product [18]. In this study, the product is physical activity and the consumers are Twitter users. We focused on eWOM rather than information sent from organizational accounts because eWOM on social networking sites features higher response rates and can be archived in a manner that extends influence to more receivers over longer periods of time compared to other marketing techniques [19]. Additionally, we were interested in the organic communication among users about physical activity, which occurs outside organizational influences and is typically the target of interventions.

To suggest guidelines for designing future health marketing interventions aiming to promote physical activity on Twitter, the current study examines both the format and the content of eWOM about physical activity on Twitter. We innovatively applied marketing and health behavior principles simultaneously. The marketing principles we examined included valence, eWOM components (ie, opinion leadership and opinion seeking), and eWOM consequences (ie, forwarding and chatting). In the context of health behavior theory, we examined physical activity modeling, social support, and negativity. Furthermore, this study examined how the characteristics of eWOM varied among tweets from users with different networking characteristics, including number of followers, number of followings (Twitter accounts that a user is following), and ratios of number of followers to followings.

### Marketing Aspects

#### Valence

Marketers are especially interested in whether their products are talked about positively, negatively, or neutrally [20]. Positive word of mouth (WOM) includes “relating pleasant, vivid, and novel experiences, recommendations to others, and even conspicuous display” whereas negative WOM includes “behaviors such as product denigration, relating unpleasant experiences, rumor, and private complaining” [21]. Exposure to negative WOM is associated with low probability of purchasing a product, whereas positive WOM is associated with high probability of purchasing [22]. For example, valence of eWOM has been found to influence box office revenue [23] and book sales [24]. Infodemiology studies have also analyzed the sentiment in surveillance of health beliefs [25] and tobacco-related tweets [12].

#### Components of Electronic Word of Mouth

In addition to valence, the current study addresses the mechanism of interactions, referred to as components of WOM [26] or eWOM [27], between Twitter users regarding physical activity. According to the path model of antecedents and consequences of online WOM, eWOM is composed of 2 forms of interactions. Online opinion leadership is the process by which people attempt to influence others’ purchasing behavior for a certain product. Online opinion seeking refers to the process by which people seek advice when purchasing a certain product [27].

A few infodemiology studies have explored how the information about different health conditions is presented on Twitter. For example, in the context of H1N1, approximately 10% of the tweets were in the form of questions [7]. In contrast, for concussions, approximately 1.4% of the tweets sought explicit advice [28]. However, little research has explored the mechanisms of interactions between Twitter users on Twitter about “purchasing” a specific health behavior (ie, physical activity participation).

#### Consequences of Electronic Word of Mouth

Based on the path model of antecedents and consequences of online word of mouth, eWOM has 2 consequences: forwarding and chatting [27]. With a limit of 140 characters (including all punctuation and spaces), Twitter is a rapid mode of communication that permits frequent updates [2]. Thus, Twitter is largely used for daily chatter, conversations, sharing information, and reporting news [2]. In addition, tweets can be archived and retrieved later by followers [29,30], extending their possible influence on others. On the other hand, the forwarding function of Twitter enables viral advertising, which is “a widely used form of unpaid communication through persuasive messages created by identifiable sponsors and distributed among peers on interactive digital platforms” [31]. Viral advertising can exponentially increase the number of people who receive a particular message and can work in conjunction with eWOM to drive communication about a topic or message.

### Health Behavior Aspects

#### Overview

Physical activity as a health behavior is a unique “product.” Purchasing in this case refers to participation. Moreover, the purchasing can be influenced by different social factors. Thus, in addition to the traditional eWOM characteristics for commercial products, this study also examined the health behavior aspects of eWOM about physical activity participation.

#### Physical Activity Modeling

Based on social cognitive theory (SCT), health behaviors can be acquired through observational learning or modeling, which is to watch and mimic the actions and outcomes of others’ behavior [32]. Observational learning can occur through many channels: face-to-face observation [32], mass media [33], and online interactions [34]. The current study examined how Twitter users provide opportunities for others to engage in observational learning about physical activity, a behavior that we refer to as physical activity modeling. In addition to actual past and current participation in physical activity, this study investigates eWOM related to the intention to participate in physical activity. Research suggests that observational learning is acquired not only from viewing others’ actions, but also through perceiving the models’ intention and then imitating their goal [35,36].

#### Social Support and Social Negativity

Apart from observational learning, SCT posits that one’s behavior is influenced by their social environment, which includes family members, friends, and acquaintances [32]. Influence exerted in a social environment can include both support and negativity [37], which have been found to play an important role in predicting physical activity participation [38,39]. Examining whether individuals exert social support and/or negativity on Twitter could provide insight into whether and to what extent Twitter might be used as a channel for social influence in future physical activity interventions. Chogahara [37] further categorized social support into 3 dimensions: companionship support, informational support, and esteem support. Chogahara also classified negativity into 3 dimensions: inhibitive, justifying, and criticizing behavior [37]. Examining these dimensions could help public health interventions target certain dimensions of social influence.

Therefore, regarding the information exchange among individuals about physical activity on Twitter, what is the distribution of (1) valence (positive, neutral, and negative); (2) eWOM components (opinion leadership and online opinion seeking); (3) eWOM consequences (chatting and forwarding); (4) physical activity modeling (communicating intention, past behavior, and current behavior); (5) social support (companionship, informational and esteem support); and (6) social negativity (inhibitive, justifying, and criticizing behavior)?

### Networking Characteristics

#### Overview

Internet social connection is an antecedent of eWOM [27]. Twitter has a unique social networking function. It enables users to choose whom to receive information from, called followings, and who can receive their information, called followers [1,2]. Both the number of followers and followings for a user are shown on the Twitter profile and can be obtained by Twitter’s application programming interface if users set their profile as public [1,2]. Thus, this study focused on 3 aspects of networking characteristics visible or easy to be estimated by other users: number of followers, number of followings, and the ratio of number of followers to followings.

#### Number of Followers and Followings

The number of contacts is an important aspect of traditional WOM [20]. The number of people following an individual is an indication of that person’s popularity on Twitter [40,41]. Popularity, in turn, is an indicator of potential influence [41]. On the other hand, the number of followings can be seen as an indicator of inquisitiveness or how much of an expert one is [42]. To guide future physical activity interventions on Twitter, the current study also explores how the number of followers and followings is associated with the way a user talks about physical activity on Twitter.

Therefore, for tweets about physical activity, how does the number of followers and followings relate to differences in (1) valence, (2) eWOM components, (3) eWOM consequences, (4) physical activity modeling, (5) social support, and (6) social negativity?

#### Followers Versus Followings

The difference between the number of followers and followings can also provide useful networking information [1,43]. Twitter users perceive other users with a narrower gap between the number of followers and followings as more credible [42]. Thus, to provide insights into designing future physical activity marketing interventions on Twitter, this study examined how the people with wider and narrower gaps between the number of followers and followings talked about physical activity differently on Twitter.

Specifically, in tweets about physical activity, how does the gap between number of followers and followings relate to differences in (1) valence, (2) eWOM components, (3) eWOM consequences, (4) physical activity modeling, (5) social support, and (6) social negativity?

##  Methods

### Data Retrieval

Using a Twitter-streaming application programming interface, 1 million tweets posted between January 1 and March 31, 2011, in the United States containing 1 of 113 key physical activity words (see Multimedia Appendix 1) in either hashtags or the text body were retrieved. The first tweet with a physical activity keyword was posted at 03:33:39 Coordinated Universal Time (UTC) on Tuesday, January 4, 2011. The millionth tweet with a physical activity keyword was posted at 00:29:34 UTC on Thursday, March 31, 2011. The keywords included all activities from lists of published physical activity measures (eg, the Physical Activity Questionnaire for adults [44], the compendium of physical activities [45], and lists of fitness programs available at a Midwestern university [46]). Synonyms were grouped after consulting a standard thesaurus and dictionaries of American slang. We also pilot-tested the keywords to ensure the list adequately addressed the physical activity content and word usage among Twitter users. To ascertain the inclusion of tweets about similar types of physical activity, different tenses, word forms (eg, walk, walking, walked), and popular Internet expressions (eg, bball and B-ball for basketball) were also used as keywords. The keywords used to search included, but were not limited to, biking, climbing, golf, hockey, jogging, pull-up, sit-up, swimming, tennis, treadmill, walking, yoga, and Zumba (see Multimedia Appendix 1).

### Scanning and Sampling

Two coders (native English speakers) were trained to scan tweets to exclude those that were not about physical activity and not from personal accounts. The exclusion criteria covered (1) tweets posted by an organization (discerned by username) and (2) tweets that included 1 of the keywords but were not about physical activity (eg, some advertisement about physical activity equipment). For example, a tweet including the word “pump” in reference to filling one’s gas tank was excluded. Non-English tweets were also excluded.

First, 30,000 tweets were randomly selected from the pool of 1 million tweets containing physical activity keywords. Second, the 30,000 tweets were sorted based on numbers generated by a random number generator. Third, the 2 coders scanned the first 16,100 tweets and yielded 4672 (29.02%) tweets that they both agreed to be about physical activity and were from personal accounts. Finally, 1500 tweets were randomly selected from the 4672 tweets (32.11%) for further coding. All 1500 selected tweets were from unique users as determined by user and Twitter account names.

###  Coding

The unit of analysis was a single tweet. The main concepts coded included (1) valence of eWOM, (2) components of eWOM,(3) consequences of eWOM, (4) physical activity modeling, (5) social support, and (6) social negativity. Coders could select all values that applied for most of the concepts except for physical activity modeling. See Table 1 for the coding scheme.

Intercoder reliability was calculated using 100 tweets. Two graduate students—a master’s and a doctoral student in public health—were trained and then completed a preliminary round of coding 100 tweets separate from the 1500 tweets. After the first round of reliability calculation, disagreements between the coders were discussed and the coding scheme was revised based on these discussions. Because some important variables were skewed (eg, showing in very few instances), this study used Holsti’s method to determine intercoder reliability [47]. The intercoder reliability scores ranged from 0.83 to 0.98 and were all acceptable (see Table 1). After the intercoder reliability was estimated, the 2 coders each coded 750 tweets.

###  Statistical Analysis

Descriptive analyses were conducted for the timing of posting the tweets, the number of followers, and people the users were following. Descriptive analyses for all eWOM characteristics were performed for (1) valence, (2) eWOM components, (3) eWOM consequences, (4) physical activity modeling, (5) social support, and (6) social negativity. Because the distribution of the number of followers and followings was quite skewed, we used the nonparametric Mann-Whitney *U* test (which does not require the normal distribution assumption) [48] to investigate if the distribution of the number of followers and followings differed across different eWOM characteristics.

For the gap between the number of followings and followers, a narrow gap was defined as a ratio of 0.9-1.1 between the number of followers and followings, whereas a wide gap group was defined as a ratio less than 0.9 or higher than 1.1 [42]. Fisher exact test was used to test if eWOM characteristics differed between the 2 groups. Fisher exact test was chosen because the data were skewed, and in most tables, 1 or more cells had expected counts less than 5 [49]. Twenty cases with zero followings were deleted, because their ratios between the number of followers and followings were not able to be calculated. Thus, the total number (N) in the analysis was 1480.

All analyses were conducted in SPSS 20 (IBM Corp, Armonk, NY, USA).

**Table 1:** Coding scheme and intercoder reliability scores of tweets about physical activity (PA).

Variables		Descriptions	Real tweet examples^a^	Reliability scores^b^
**Valence (select all that apply)**				
		Explicit sentiments associated with either a kind of PA itself or participating in PA or the environment where PA takes place		
	Positive	Complimenting and relating pleasant and vivid experiences; praise/favorableness	A best day of personal fitness EVER=running around kinnick. :) football season get here fasterrrr	0.91
	Negative	Complaining and the relating of unpleasant experiences	No one wants to go to the rec with me... #wah	0.96
	Neutral	No sentiment	fooood, cleaning, gym...	0.88
**Components of eWOM about PA (select all that apply)**				
	Online opinion leadership	Giving out information or opinions about PA (including PA itself or participating in PA or the environment where PA takes place)	Morning jog. Tennis later. We love sports!	0.97
	Online opinion seeking	Asking for information and opinions of PA (including PA itself or participating in PA or the environment where PA takes place)	Shall I go swimming or take a bangin nap when i get off work in 30mins?	0.97
**Communication consequences of eWOM (select all that apply)**				
	Online chatting	Provide plain text	Finally gym time. If anyone see&apos;s JLove on Michigan Ave. Tell her I said no to the pretty red sole shoes in the window!!	0.94
	Online forwarding	Forward what other people said or the content of a Web page about PA. It could include sharing the Twitter messages from other Twitter users. When the Twitter message contains RT,” code all the contents before and/or following RT. Forwarding the Twitter messages from Web, when the Twitter message contains a website link (URL). If a tweet includes a link, do not need to analyze the content in the link. But you can follow the link to help understanding	RT @* Im bout to go swimming...	0.94
**PA modeling (single choice)**				
		Either ones’ own experience or others experience including previous experience and intention to future experience		
	Intention to participate in a PA	A statement showing the participant is going to or needs to participate in PA (including intent to and nonintent to)	Feels like going to the gym	0.83
	Past experience of participating in a PA	A statement that they have done a PA but did not give any advice or support to others	Went to the gym. Tired. Hanging out at older sis house now. Nap is probably needed	0.86
	Current status of participating in a PA	A statement indicating the participation in PA right at the moment when posting the messages	@*still running :)) 2 more km. .and you? Are you still hungry? :D	0.93
**Social support and social negativity (select all that apply)**				
		Not about oneself; need another person involved		
	Companionship support	Partnership assistance of a PA that suggests “we participate together”(components: coplanning, cooperation, coparticipation, reminding, rescheduling, offering, willingness)	Went on an enjoyable run with the lovely *. Now to do homework for the rest of the night...	0.86
	Informational support	Knowledge assistance of a PA that suggests “you should know” (components: enlightenment, rationalization, clarification, program referral, intensity suggestion, activity recommendation, supporter referral, problem solving, and goal direction)	@* I recommend it for a beginners workout. . DM and I can give you more information.	0.97
	Esteem support	Esteem information provision of a PA that suggests “you are good” (components: mastery recognition, social comparison, affirmation, respect, reinforcement, interest, and reassurance)	@LAEasyMeals Congrats! Can&apos;t imagine running 26.2 in that heat but well done!	0.97
	Inhibitive behavior	Disapproval and discouraging behavior that suggests “you should not participate in PA” (components: warning, delimitation, worrying, forbidding, threatening, disapproving, and rejection)	@* hott as hell in southeast gym	0.98
	Justifying behavior	Excusing and overprotective behavior that suggests “you don’t need to participate in PA” (components: excuse-giving, compromising, exempting, pardoning, and ignoring)	“Girl! YOUR body is a solid A++++++++ you don’t need to work out!!” - @*	0.95
	Criticizing behavior	Demanding and blaming behavior that suggests “you are not good at doing PA” (components: exclusion, demanding, nagging, contempt, bothering, depressing, and ridicule)	I’ve been told I play basketball like a girl haha...	0.97

^a^Personal names replaced with * to maintain confidentiality.

^b^Using Holsti’s method (n=100).

## Results

### Overview

**Figure 1:**
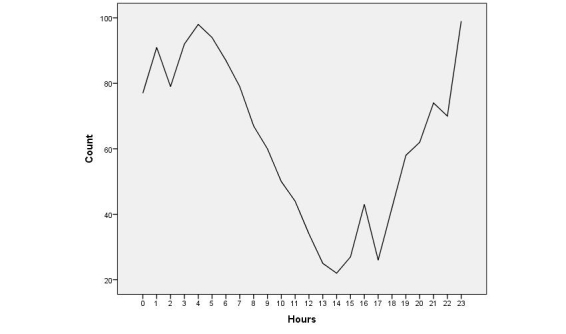
Hourly distribution of all 1500 tweets during the day from hour 0 (00:00-00:59) to hour 23 (23:00-23:59) Coordinated Universal Time (UTC).

All 1500 tweets were posted by 1500 distinct users in the first quarter (January 1 to March 31) of 2011 in the United States. Tuesday was the most popular day for posting (245/1500, 16.33% of posts) and followed closely by Monday (234/1500, 15.60%), Wednesday (228/1500, 15.20%), and Thursday (227/1500, 15.13%). Friday was least popular (182/1500, 12.13%), followed by Sunday (184/1500, 12.27%) and Saturday (200/1500, 13.33%). Figure 1 presents a snapshot of the hourly distribution of all 1500 tweets from 0 (00:00-00:59) to 23 (23:00-23:59) UTC.

**Figure 2:**
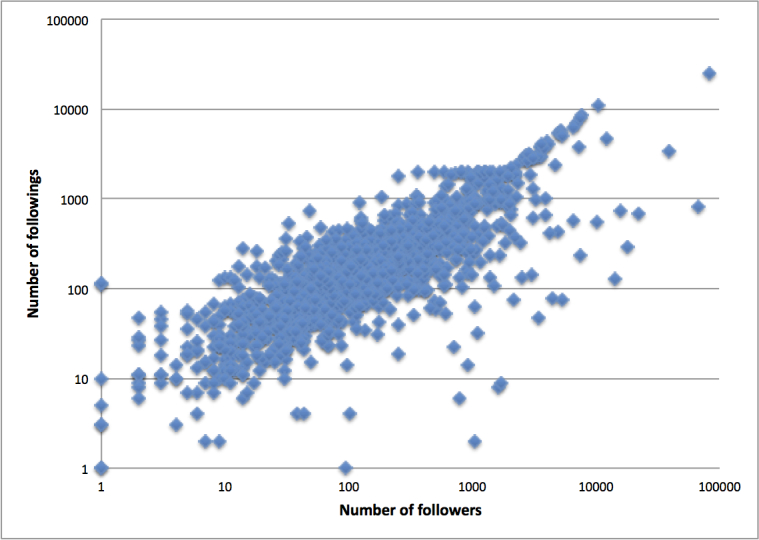
Scatterplot of the number of followers and followings of users (n=1472).

Users had an average of 576 followers (SD 3183, range 0-82,874, median 122). Users followed an average of 368 people (SD 976, range 0-25,069, median 148). Users posted 6630 total tweets on average, ranging from 1 to 167,517 (SD 14,158). Figure 2 presents the relationship between number of followers and followings with the axes in logarithmic scales among the 1472 Twitter users. We excluded 28 users who did not have any followers or followings from the 1500 Twitter users.

###  Descriptive Distribution

Table 2 presents the numbers of tweets in each category (eg, positive) of eWOM characteristics (eg, valence). Because 1 tweet can present in more than 1 category of each characteristic (except for physical activity modeling), the numbers in the categories of each eWOM characteristic (except for physical activity modeling) are not mutually exclusive.

Regarding the distribution of eWOM valence (ie, positive, neutral, or negative), approximately 85% of the tweets (1270/1500, 84.67%) had neutral valence only. Tweets with only negative valence comprised less than 3% (41/1500, 2.73%) of the total. In addition, there was 1 tweet (1/1500, 0.07%) that had both positive and neutral value, 1 (1/1500, 0.07%) that had both negative and neutral value, and 4 (4/1500, 0.27%) that had both positive and negative values.

Regarding the distribution of online opinion leadership and online opinion seeking, nearly all tweets (1464/1500, 97.60%) illustrated online opinion leadership only. Online opinion seeking alone was rare (26/1500, 1.73%). In addition, 10 tweets (10/1500, 0.67%) performed both online opinion leadership and opinion seeking.

For the 2 consequences of eWOM (online chatting and online forwarding), approximately 9 in 10 tweets (1354/1500, 90.27%) were in the form of online chatting only. Online forwarding alone occurred in about 1 in 13 tweets (108/1500, 7.20%). Another 36 tweets were in the both forms of online chatting and forwarding.

For the distribution of physical activity modeling, including communicating intention to participate in physical activity, past experience participating in physical activity, and current participation (and intention to participate) in physical activity, approximately 60% (905/1500, 60.33%) of tweets were related to 1 of these 3 areas. Of the tweets that mentioned intention or behavior, more than half (469/905, 51.82%) were about past behavior.

Regarding the distribution of different dimensions of social support, more than 90% of the tweets did not mention any social support (1364/1500, 90.93%). Among the tweets that offered social support (135/1500, 9.00%), informational support was the most frequent (63/135, 46.67%). Social negativity occurred in less than 2% of the tweets (18/1500, 1.20%).

###  Association Between Number of Followers and Electronic Word of Mouth

The number of followers differed between tweets with positive valence (n=186, mean 508, SD 2919) and others (n=1314, mean 586, SD 3219; *P*=.02). The number of followers also differed between tweets with neutral value (n=1272, mean 595, SD 3270) and others (n=228, mean 473, SD 2645; *P*=.04) in response to valence. There were no significant associations between number of followers and eWOM components, eWOM consequences, physical activity modeling, social support, and social negativity.

### Association Between Number of Followings and Electronic Word of Mouth Characteristics

We explored the association between the number of followings and the different aspects of eWOM (see Table 2). The number of followings differed between tweets with positive value (n=186, mean 283, SD 506) and others (n=1314, mean 380, SD 1025; *P*=.04).

There were no significant associations between number of followings and eWOM components, physical activity modeling, social support, and social negativity. The number of followings differed between users who forwarded tweets about physical activity (n=139, mean 375, SD 814) and others (n=1361, mean 367, SD 992; *P*=.04). In summary, people who talked positively about physical activity were likely to follow fewer people, whereas people who forwarded information about physical activity were likely to follow more people.

### Gaps Between Number of Followers and Followings and Electronic Word of Mouth Characteristics

There were no significant associations between the gap between numbers of followers and followings and valence, eWOM components, eWOM consequences, physical activity modeling, and social negativity. For social support, it was found that people with a wider gap between the number of followers and followings were more likely to provide companionship (*P*=.04). Table 3 presents the distribution of companionship support based on the gaps between number of followers and followings (OR 0.31, 95% CI 0.10-1.0).

**Table 2:** Descriptive electronic word of mouth (eWOM) characteristics of tweets about physical activity (PA).

Characteristics of eWOM			n^a^	Number of followers Mean (SD)	Number of followings Mean (SD)
**Valence**					
	Positive		188	508 (2919)^b^	283 (506)^b^
	Negative		46	302 (517)	308 (521)
	Neutral		1272	595 (3273)^b^	382 (1039)
**Components of eWOM**					
	Online opinion seeking		36	383 (657)	332 (456)
	Online opinion leadership		1474	583 (3220)	370 (986)
**Consequences of eWOM**					
	Chatting		1390	582 (3316)	367 (993)
	Forwarding		144	535 (1478)	375 (814)^b^
**PA modeling**			905		
	Intention		336	397 (1381)	278 (469)
	Past behavior		469	600 (2474)	325 (604)
	Current behavior		100	336 (636)	326 (566)
**Social support and negativity**					
	**Social support**				
		Companionship support	51	440 (827)	310 (560)
		Informational support	63	1899 (8504)	900 (1952)
		Esteem support	23	420 (971)	193 (168)
		None	1364	523 (2780)	348 (922)
	**Social negativity**				
		Inhibitive behavior	10	1354 (1773)	377 (384)
		Criticizing behavior	13	128 (177)	141 (166)
		None	1481	577 (3201)	368 (981)

^a^The n in categories of each eWOM characteristics (except for PA modeling) were not mutually exclusive.

^b^ *P*<.05.

**Table 3:** Companionship support and gaps between number of followers and followings.

Gaps between number of followers and followings	Companionship support, n (%)		Total, n
	Yes	No	
Narrow gap (0.9-1.1)	3 (1.2)	240 (98.8)	243
Wide gap (<0.9 or >1.1)	48 (3.9)	1189 (96.1)	1237
Total	51 (3.4)	1429 (96.6)	1480

##  Discussion

### Principal Findings

This exploratory study examined whether and how people talk about physical activity on Twitter. First, this study examined the valence of physical activity eWOM, an important marketing concern. Second, it explored the components of the path model of antecedents and consequences of online word of mouth [27]. Third, it addressed 3 important constructs of SCT [32]: observational learning (physical activity modeling in this study), social support, and social negativity. Finally, this study tested for differences in eWOM characteristics associated with the number of followers, followings, and the ratio of number of followers to followings.

The distribution of valence across physical activity tweets was different from other commercial products. In a study examining tweets for a variety of products (eg, automotive, computer hardware, consumer electronics, energy, fast food, Internet services, personal care, sporting goods, and transportation), approximately 60% of the tweets were positive, 12% were neutral, and 23% were negative [1]. One recent content analysis on tobacco-related tweets found more positive than negative or neutral tweets [12]. However, in the current study, most tweets (85%) were neutral. This finding reflects a possible difference in eWOM between tangible commercial products and health behavior. For instance, commercial products involve tangible costs and benefits, and the transaction can be completed relatively easily in a short time. Physical activity involves more intangible costs, including time and energy, and usually takes longer to “consume.” Moreover, the potential benefits of engaging in physical activity can take even longer to observe. This may preclude users from commenting positively or negatively about physical activity. An alternative explanation is that people might be less willing to comment or have more difficulty commenting on their own behaviors than on commercial products. When people comment on a product or service, they evaluate third-party providers, which is a relatively easy task. When discussing physical activity, however, they have to evaluate their own behaviors and their own selves, which may be more difficult cognitively.

The number of positive physical activity tweets was 4 times higher than negative physical activity tweets. This finding is consistent with past literature, which has shown that positive WOM was more common than negative WOM in 15 studies, with an average incidence ratio of 3:1 [50].

For eWOM components, the results of this study indicate that Twitter is currently used more often to provide opinions or information than to seek opinions or information about physical activity. This finding is consistent with a content analysis of tweets about concussions, in which researchers found that only approximately 1.4% of tweets sought explicit advice [28]. Our finding also suggests that posting public messages on Twitter is not yet a popular method for seeking physical activity information or opinions. The low percentage of tweets seeking opinion or information might indicate that people are using more traditional WOM communication or other kinds of eWOM channels to seek information. People could also be sending direct tweets, which are private between 2 users, to seek opinions and/or information about physical activity.

Regarding eWOM consequences, chatting was more common than forwarding among the physical activity tweets, a finding consistent with a previous study that found that one of Twitter’s main functions was daily chatter [2]. It is also consistent with the primary usage of social network sites for health information: health updates and queries [13].

Physical activity modeling was represented in more than half of the tweets (60%). This finding is not surprising because the Pew Internet & American Life Project found that among 27% of Internet users the most common use of online health communication was to track weight changes, manage diets, record exercise routines (which could qualify as physical activity modeling), or follow some other health indicators or symptoms [13]. This finding is also consistent with a previous study regarding concussion reporting on Facebook: most of the posts (65%) shared a personal experience [51].

In addition to examining how people might model physical activity in their tweets, this study is the first to examine both social support and negativity via eWOM on Twitter. Given that eWOM about daily routine is the most common use of Twitter [2], our finding that only 10% of tweets provided any kind of social support or negativity is not surprising. The results of this study suggest that Twitter is currently not a popular platform for social influence attempts regarding physical activity. However, people could be using direct tweets to ask for or to provide their followers with social support. These direct, private tweets were not available for this study. Future research might incorporate those tweets to examine how people perceive social support or social negativity and these messages are influential. On the other hand, another popular social networking site, Facebook, has been linked to social support among users [52]. This may indicate that certain characteristics of Twitter make it an unlikely place to seek and obtain social support, unlike other online platforms, such as Facebook or discussion groups. Such features could be Twitter’s immediacy and the forced brevity of the updates (only 140 characters).

Regarding the association between the number of followers and followings and the eWOM characteristics, the results suggested that people with fewer followers and followings were more likely to talk positively about physical activity on Twitter. People with more followers were more likely to post neutral tweets about physical activity. People with more followings were more likely to forward tweets. These findings suggest that people with different number of followers and followings may have different motivations for using Twitter regarding physical activity. People with fewer followers and followings might be more likely to connect with a close social network on Twitter and talk about physical activity positively for fun, whereas people with more followers and followings might be more likely to use Twitter primarily for information sharing about physical activity. However, future research is needed to further examine the reasons and confirm these suggestions.

Finally, a surprising finding is that people who had a wider gap between the number of followers and followings were more likely to mention companionship support on Twitter. This contradicted the intuition that a narrower gap between the number of followers and followings might indicate higher reciprocity between actual friends, which could result in more mentioning of social support on Twitter. This result can be explained by the finding from another study that Twitter is a sparse network for actual friends rather than a dense network between followers and followings [53]. Only approximately one-third of the users on Twitter are followed by their followings [1]. So the difference between number of followers and followings might not reflect the number of actual friends. It could be possible that people who have a wider gap between number of followers and followings might have more actual friends on Twitter to whom they provide companionship support.

There could be other alternative explanations. For example, because companionship support of physical activity requires the geographic accessibility and proximity of 2 people or more, the offline relationship between the users is indispensable. Thus, people with a narrower gap between number of followers and followings might be receiving and offering companionship support through other offline channels, such as face-to-face, telephone, text messaging, or even through direct tweets between one another that are private and, thus, could not be retrieved in our study.

###  Practical Implications

Considering the low prevalence of positive tweets in contrast to the high proportion of physical activity modeling, future interventions should encourage people not only to chat about their physical activity intention or participation, but also to express the benefits of physical activity and their positive experiences with it.

Examining tweets for the components of SCT suggests that Twitter is currently mostly used for general observational learning of physical activity instead of exerting social support or social negativity. More innovative methods, such as infovigil robot, can be used to direct Twitter users to social support interventions after they post any tweets about physical activity [3]. In addition, examining tweets based on the path model of antecedents and consequences of online WOM [27] can inform public health practitioners about specific communication strategies that can be used to promote physical activity on Twitter. Future interventions could encourage Twitter users to provide opinion or information about physical activity through chatting because this study found that most tweets were examples of opinion leadership rather than opinion seeking.

Findings about how eWOM characteristics differed among Twitter users with different networking characteristics can provide insights into segmentation of audiences in future physical activity marketing interventions on Twitter. The association between the number of followers and followings and the valence of eWOM about physical activity indicates that interventions encouraging positive discussion of physical activity could start by enrolling individuals with fewer followers and followings and observing and learning how they talk positively about physical activity.

Because people with more followings tended to forward opinions or information about physical activity on Twitter suggests that public health practitioners could target people with more followings in future physical activity marketing interventions. Public health practitioners could develop Twitter accounts to promote physical activity and encourage Twitter users to follow the accounts and retweet tweets about physical activity to their followers.

###  Limitations and Future Research

The first limitation of this study is associated with the sampling method. This study used a list of keywords to retrieve tweets about physical activity. Although a comprehensive list of keywords was generated based on the physical activity checklist of the Physical Activity Questionnaire (Adults) [44], the compendium of physical activities developed by Ainsworth et al [45], and the list of fitness programs and intramural sports at the Midwestern university where this research was conducted [46], the possibility that some of the keywords were not captured (eg, the words with hierarchical relationships) cannot be overlooked. Future study should explore more vocabulary for keywords and use query expansion techniques to group similar keywords.

A second limitation relates to the lack of demographic information about the Twitter users. This limitation has also been observed in other studies [7]. Nevertheless, determining differences in talking about physical activity on Twitter based on different demographic characteristics, such as age and race, was not possible in this study. Future research might obtain permission from Twitter users to collect their demographic information. Moreover, future study could retrieve user-aggregated data and perform studies on the scale of individual users [6].

A third limitation is the lack of information about other social network characteristics of the Twitter users. Future studies would benefit from collecting information about reciprocal followings, which indicates the potential for interactive communication between users and their followers [54]. Moreover, this study used only the number of followers as an indicator of influence. Other indicators of influence were beyond the scope of the study; for example, neither message value (measured by the frequency of tweets passed between users) nor name value (measured by the frequency with which a name is mentioned in tweets from other users) was measureable in this study [41]. Future research can retrieve more information about message value and name value to help identify opinion leaders of physical activity and to investigate how they talk about physical activity on Twitter. Future study can also examine the social circles about physical activity on Twitter, adapting the method of a recent study on social circles about prescription drug abuse on Twitter [10].

Fourth, considering the effects of social support on individuals’ physical activity based on SCT [32], future formative research using qualitative methods, such as interviews or focus groups, is needed to explore the predictors and barriers of using Twitter as a social support platform. The results could guide public health practitioners to develop interventions that encourage people to provide more social support via eWOM about physical activity on Twitter.

Although the tweets selected represented a random sample, the number of tweets about physical activity retrieved and coded was limited. First, because of the huge number of tweets posted every day, this study could not retrieve tweets in the United States for an entire year to control for physical activity variation in different seasons. We only included the tweets in the first quarter of the year. Second, although human coding enables the accurate categorization for different characteristics of eWOM at the same time, because of the labor intensity in manual coding, 2 coders only coded 1500 tweets in this study. Future study could use crowdsourcing experiments to conduct large-scale studies to provide a broader picture of the eWOM about physical activity on Twitter in the United States. Guided by the coding scheme invented and tested in this study, future studies could also explore a machine learning application and compare human coding and computer coding. With a larger number of tweets, future study could also localize different physical activities and physical activity eWOM by geographic region [5,6].

###  Conclusions

This study is the first to examine the content of eWOM about physical activity on Twitter. Twitter demonstrated potential for chatting and physical activity modeling (ie, intention, past behavior, and current behavior), as well as talking neutrally about physical activity. To guide future physical activity marketing interventions on Twitter, this study also provides insights into segmenting audiences based on user profile information about number of followers and followings. Having more followings was associated with forwarding information. Having fewer followers and fewer followings was associated with talking positively about physical activity. Having more followers was associated with talking neutrally about physical activity. Having a wider gap between the number of followers and followings was associated with mentioning companionship social support about physical activity on Twitter. Future studies could apply the innovative perspectives from marketing and public health used in this exploratory study for larger-scale infodemiology studies that could also examine tweets about physical activity as well as other health behaviors.

## Multimedia Appendix 1

Physical activity keywords.

## Abbreviations

eWOM: electronic word of mouth
PA: physical activity
SCT: social cognitive theory
WOM: word of mouth
