# Supplementary material for: Electronic Word of Mouth on Twitter About Physical Activity in the United States: Exploratory Infodemiology Study
Source: J Med Internet Res. 2013 Nov 20;15(11):e261. doi: 10.2196/jmir.2870 (PMC3841353; doi:10.2196/jmir.2870)
Supplement: Supplementary file 1 [file jmir_v15i11e261_app1.pdf]

APPENDIX PHYSICAL ACTIVITY KEYWORDS

Aerobics	Aikido	badminton	balanced	barbell	baseball	basketball
baton Twirling	BB	bball	B-ball	bicycle	bicycling	bike
biking	boat	bowling	canoe	canoeing	cardio	circuit
climb	climbed	climbing	cross county	Cy-Yo_Thom	dance	dancing
dodgeball	dumbbell	elliptical	exercise	fitness	football	Frisbee
golf	gym	gymnastics	Hapkido	hike	hiked	hiking
hockey	hunt	hunting	interval	Jazzercise	jog	jogged
jogging	Judo	Karate	kayak	kayaking	Kickball	Lacrosse
martial	muscle	paddle	paddling	physical activity	pull-up	pump
pumping	push-up	Racquetbal l	ran	rec	recreation	resistance
rock	row	rowing	run	Salsa	shoot	shooting
sit-up	skate	skating	ski	skiing	soccer	softball
squash	squats	step	strengthen	stretch	stretching	swim
swimming	table tennis	Tae Kwon Do	tennis	track & field	treadmill	ultimate
upper class	VB	vball	v-ball	volleyball	walk	walked
walking	water	weight	wiffleball	workout	Wrestling	Yoga
zumba						
